# Genome-Wide Characterization and Analysis of the *SPL* Gene Family in *Eucalyptus grandis*

**DOI:** 10.1155/2024/2708223

**Published:** 2024-09-11

**Authors:** Lijun An, Jiasi Ma, Chunjie Fan, Huiling Li, Aimin Wu

**Affiliations:** ^1^ Guangdong Key Laboratory for Innovative Development and Utilization of Forest Plant Germplasm College of Forestry and Landscape Architectures South China Agricultural University, Guangzhou 510642, China; ^2^ State Key Laboratory of Tree Genetics and Breeding Key Laboratory of State Forestry and Grassland Administration on Tropical Forestry Research Institute of Tropical Forestry Chinese Academy of Forestry, Guangzhou 510520, China

**Keywords:** bioinformatics analysis, *E. grandis*, gene expression, *SPL* gene family

## Abstract

SQUAMOSA promoter-binding protein-like (*SPL*) gene family, a group of plant-specific transcription factors, played crucial roles in regulating plant growth, development, signal transduction, and stress response. This study focuses on the *SPL* gene family in the fast-growing *Eucalyptus grandis*, employing bioinformatics approaches to identify and analyze the gene physiochemical characteristics, conserved domains, structural composition, chromosomal distribution, phylogenetic relationships, cis-acting elements, and their expression patterns in various tissues and stress treatments. Twenty-three *SPL* genes were identified in *E. grandis*, which uneven distributed across seven chromosomes and classified into five groups. Prediction of cis-acting elements revealed that these genes might be related to light, hormone, and stress responses. Furthermore, *EgSPL9* and *EgSPL23*, mainly expressed in the stem apex and lateral branches, seem to be involved in hormone stress resistance. Our study provides insights into the potential functions of the *EgSPL* genes in plant growth, stress response, and hormone transduction, offering valuable perspectives for subsequent research into their biological roles.

## 1. Introduction

The SQUAMOSA promoter-binding protein-like (*SPL*) contained a highly conserved squamous promoter-binding protein (*SBP*) domain consisting of 76 amino acid residues and two specific zinc finger motifs (composed of 8 cysteine (Cys) or histidine (His) residues) [1]. As the plant-specific transcription factor family, it was first discovered in *Arabidopsis thaliana* [2] and was able to specifically recognize the SQUAMOSA (SQUA) promoter. Consequently, the *SPL* transcription factors play important roles in multiple stages of plant growth and development including initiation of flowering [3, 4], stem and leaf development [5, 6], flower and fruit formation [7], male sterility, and response to abiotic stress [8–10].

In plant, *SPL* genes showed diverse and critical roles for various developmental and physiological processes. For instance, *AtSPL2*, *AtSPL10*, *AtSPL11*, and *AtSPL13* are involved in regulating transition from vegetative growth to reproduction growth [11]. *AtSPL3* and its homologous genes *AtSPL4* and *AtSPL5* are involved in flowering [12, 13]. *AtSPL9* and *AtSPL15* mainly regulated leaf growth and the initiation of flowering [14] while they also were explored involving in the transition from juvenile to adult developmental stages [15–17]. On the contrary, *AtSPL14* seems to have a function in delaying phase transition [18]. Moreover, comprehensive studies have revealed that most *SPL* family members harbor the response target element of *miR156*, a type of noncoding RNA predominantly involved in regulating gene expression at the posttranscriptional level [19]. Specifically, in *A. thaliana*, the mutation of the *miR156* binding site impedes its negative regulation of *AtSPL13,* significantly delaying the appearance of the leaf primordium and therefore delaying the growth of true leaves during germination [20]. *AtSPL8* cooperated with other *SPL* genes with *miR156* target sequence, which further regulate early anther development and pistil differentiation, as well as sepal epidermal trichome formation, root growth, microsporogenesis, and megasporogenesis [21–24].

Additionally, *SPLs* also played an important role in stress resistance. *AtSPL7* regulates copper (Cu) homeostasis and cadmium (Cd) stress [25, 26]. The *AtSPL10* binds to and activates key defense genes, thereby enhancing resistance in *A. thaliana* leaves [27]. Under conditions of NaCl stress, the expression of the *BpSPL9* gene in birch roots and leaves is upregulated, facilitating the scavenging of reactive oxygen species [9]. Tea *CsSPL1* and *CsSPL12* were inhibited by abscisic acid (ABA), GA, and salt treatment and induced by ethylene [28].

Since the functional importance of *SPL* genes, researchers have developed genome-wide identification of *SPL* genes in numerous plant species. There was 17 *SPL* genes identified in *A. thaliana* [29], 28 *SPL* genes in *Populus trichocarpa* [30], 21 *SPL* genes in *hybrid poplar* [31], and 18 *SPL* genes in *Betula luminifera* [32]. Although *SPL* genes in the same evolutionary branch have certain similarities, many *SPL* genes from the same branch might have different functions in different plant species as well.

*Eucalyptus grandis*, distinguished by its rapid growth rate and widespread geographical distribution, is highly valued for its superior wood and fiber qualities. Completeness of genome sequence of *E. grandis* provided an opportunity to elucidate the molecular mechanisms governing its growth, development, and response to environmental stressors. Currently, the biological role and precise molecular mechanism of the *SPL* genes were elusive. Comprehensive annotation of *EgSPLs* is the first step towards elucidation of the underlying molecular mechanisms. This study conducted a genome-wide analysis of the *E. grandis SPLs*, including the systematic identification of conserved structure, phylogeny, chromosomal location, collinearity analysis, promoter homeopathic element analysis, and its expression in various tissues and different stress conditions. Our study lays the foundation for exploring functions of *SPLs* in transcriptional regulation of *E. grandis*.

## 2. Materials and Methods

### 2.1. Identification and Physicochemical Properties

We acquired genome-wide data of E. grandis from E. grandis Genome Database (https://phytozome-next.jgi.doe.gov/info/Egrandis_v2_0), and downloaded 17 A. thaliana AtSPL gene sequences from the TAIR database (https://www.Arabidopsisthaliana.org/browse/genefamily/sbp_box_genefamily.jsp). Through blast comparison, initial screening is conducted to obtain *EgSPL* family–related genes. Utilizing the NCBI CDD online tool to predict SPL protein domains, excluding atypical domains and incomplete redundant sequences, the *EgSPL* family members are identified. Analysis of the physicochemical properties of EgSPL proteins is performed using the online software ExPASy ProtParam (https://web.expasy.org/protparam/), the subcellular localization of *EgSPL* genes was predicted using the Plant-mPLoc platform (http://www.csbio.sjtu.edu.cn/cgi-bin/PlantmPLoc.cgi), and potential *miR156* target sites within the *EgSPL* genes were identified through predictions made on the psRNA target online platform (http://plantgm.noble.org/psrnatarget/).

### 2.2. Phylogenetic Tree Analysis

To elucidate the evolutionary relationships among *SPL* genes across diverse plant groups, including algae, ferns, dicots, and woody plants, the protein sequences of *Physcomitrium patens*, *Selaginella moellendorffii*, *A. thaliana*, and *E. grandis* were selected for comparison analysis. The resulting data were analyzed using the maximum likelihood (ML) method implemented in the TBtools software [33], with bootstrap analysis set to 2000 replicates, to construct a comprehensive phylogenetic tree. Lastly, the phylogenetic tree file was input into the Evolview website (https://www.evolgenius.info/evolview/) for annotation and optimization.

### 2.3. Conservative Domain and Genetic Structure Analysis

The conserved motifs of the EgSPL proteins were analyzed using the MEME online software (http://memesuite.org/tools/meme), with the number of identified motifs set to 10. The results of this analysis were saved in an XML file format, which was subsequently visualized using TBtools [33]. Additionally, the gene structure of the *EgSPL* gene family was elucidated through analysis in TBtools software, utilizing the gff3 file of the *E. grandis* genome.

### 2.4. Chromosome Positioning and Collinearity Analysis

The gff3 file in the *E. grandis* genome was used in TBtools software to obtain the staining and positioning information of the *EgSPL* family members and draw the distribution map of the genes on the chromosome. Genome sequences of *E. grandis*, *A. thaliana*, and *P. alba* were downloaded from the Phytozome (https://phytozome-next.jgi.doe.gov/). Genome collinearity analysis was conducted using the One Step MCScanX function in TBtools software, focusing on the species *E. grandis* and its comparative analyses with *A. thaliana* and *P. alba*. The parameter “Num of BlastHits” was set to 4 for the interspecific comparisons, and the *E*-value was set to 1e−10.

### 2.5. Cis-Element Analysis of EgSPLs Promoters

The upstream 2000 bp sequence of *SPL* genes as promoter was extracted by using TBtools, and PlantCARE (https://bioinformatics.psb.ugent.be/webtools/plantcare/html/) was applied to predict the cis-acting elements. The predicted cis-elements were processed and visualized by using Excel and TBtools, respectively.

### 2.6. Plant Material and Gene Expression Pattern Analysis and Growth Conditions

This tissue from *E. grandis* trees was from 6-month-old saplings providing roots, leaves, xylem, phloem, and stem internodes, while mature trees (3 and 6 years old) supplied organs such as flowers, xylem, phloem, and cambium. Hormonal treatments with salicylic acid (SA) or methyl jasmonate (MeJA) and salinity stress were applied to 2-month-old shoots. Samples were collected at 0, 1, 6, 24, and 168 h posttreatment. All experiments were performed triple replicates at least, ensuring a robust dataset for subsequent analysis. The detail transcriptomic data for *E. grandis*, detailing gene expression across tissues and following treatments with SA, MeJA, and salt [34], were retrieved from a dedicated eucalyptus database (https://bar.utoronto.ca/%7Easher/eplant_eucalyptus/). Subsequent to data extraction, heat maps were generated using TBtools to visually delineate and interpret the expression profiles, offering a comprehensive overview of the gene activity under various physiological and stress conditions.

### 2.7. Prediction of Protein Structure

The three-dimensional structure of EgSPL protein was predicted through the SWISS-MODEL website (https://swissmodel.expasy.org/). Upload the amino acid sequence to the website, select the model with the highest matching degree, among which the similarity of the selected models is above 40%, and output the file for analysis.

## 3. Results

### 3.1. Identification and Physicochemical Properties

A total of 23 members of the *SPLs* were identified by removing proteins without conserved domains from the genome of *E. grandis*. According to the result from two-way blast comparison, these genes were named *EgSPL1-23* and the details are listed in Table 1. And their physiochemical properties are also shown in Table 1. The lengths of EgSPL proteins are 147–1078 aa (no. of amino acids), with a relative molecular weight (MW) ranging from 19.95 to 119.29 kDa (molecular mass). The isoelectric points (pI) of the EgSPL proteins range from 5.79 to 9.50, with *EgSPL1*, *EgSPL4*, *EgSPL9*, *EgSPL16*, and *EgSPL22* having values less than 7, classifying them as acidic proteins. The instability index of EgSPL protein ranged from 50.07 to 88.37, of which *EgSPL2* was 88.37, was most instable protein. In addition, the grand average of hydropathicity of all EgSPL protein was negative, which ranged from −1.44 to −0.41. Among the 23 *EgSPL* genes, 16 genes contain target sites for *miR156*. However, there are still seven genes including *EgSPL3*, *EgSPL9*, *EgSPL10*, *EgSPL12*, *EgSPL20*, *EgSPL22*, and *EgSPL23*, which were not identified targeting sites of *miR156*. Most of the *EgSPL* genes were located in the nucleus, and only a few genes, including *EgSPL1*, *EgSPL4*, *EgSPL14*, *EgSPL16*, and *EgSPL22*, appeared in both the nucleus and the cytoplasm (Table 1).

### 3.2. Phylogenetic Analysis

**Figure 1:**
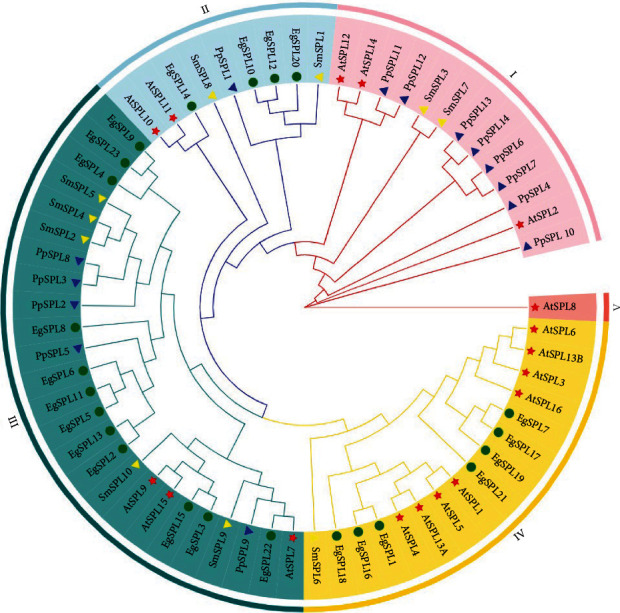
Phylogenetic analysis of *SPLs* from *E. grandis*, *A. thaliana*, *S. moellendorffii*, and *P. patens*. The ML tree was constructed using TBtools software with 2000 bootstrap replicates. Signs of different shapes represent *SPL* genes from *E. grandis* (green round, Eg), *A. thaliana* (red star, At), *S. moellendorffii* (yellow triangle, Sm), and *P. patens* (blue triangle, Pp).

To elucidate the evolutionary relationships of *SPL* genes in plants, we constructed a phylogenetic tree using SPL protein sequences from *E. grandis*, *A. thaliana*, *S. moellendorffii*, and *P. patens*, which were divided into five groups (Figure 1). Notably, no *EgSPL* genes existed in Group I, raising questions about potential gene loss in the evolutionary trajectory of the *E. grandis* genome. In Group III, *EgSPL* genes form a close cluster with *SPL* genes from *S. moellendorffii* and *P. patens*, suggesting a shared common ancestor during evolutionary processes. In Group IV, a pronounced clustering of *SPL* genes from both *A. thaliana* and *E. grandis* is observed, suggesting a more recent common evolutionary lineage or a closer phylogenetic relationship between these genes from the two species. Specifically, *EgSPL3* and *EgSPL5* align with *AtSPL9* and *AtSPL15* from *A. thaliana*, respectively. The convergence of *EgSPL22*, *AtSPL7*, *PpSPL9*, and *SmSPL9* on the same branch is particularly intriguing and warrants further investigation to elucidate any potential functional similarities or shared evolutionary trajectories. Further analysis revealed that the number of *SPL* family genes has increased and gene duplications have been significantly enhanced from lower to higher plants, reflecting the evolutionary success of flowering plants in adapting to diverse and fluctuating environmental conditions [35].

### 3.3. Gene Structure and Conserved Motif Analysis

**Figure 2:**
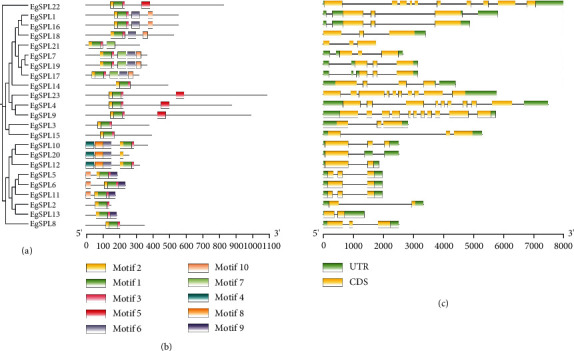
Analysis of conserved motifs and gene structure of *SPL* gene family members in *E. grandis*. (a) TBtools are used to construct the evolutionary tree of 23 *EgSPL*. (b) The conserved motifs of the EgSPL proteins predicted on MEME. (c) The structure of the *EgSPL* genes is visualized based on gff file. Exons, introns, and UTRs are represented by yellow square rectangles, black lines, and green square rectangles, respectively.

Evolutionary analysis of *EgSPL* genes suggests that genes from the same evolutionary branch often have similar conserved domains, regardless of the closeness of their relationship (Figure 2(a)). Conserved domain analysis of the EgSPL proteins indicates that all members include Motifs 1, 2, and 3, suggesting that these are fundamental to *SPLs* function (Figure 2(b)). Specific structural domains were also identified, such as Motifs 4 and 8 existed in *EgSPL10*, *EgSPL12*, and *EgSPL20* suggesting the possibility of their functional attributes and performing similar biological roles. Gene structure analysis illustrates variation in exon–intron configurations among the *EgSPL* family members, with the number of exons ranging from 2 to 11 (Figure 2(c)). Furthermore, *EgSPL9* has an 11-exon structure, the most among all *EgSPL* genes. Moreover, genes that are phylogenetically closed often show similar intron–exon structures.

### 3.4. Chromosomal Locations and Synteny Analysis

**Figure 3:**
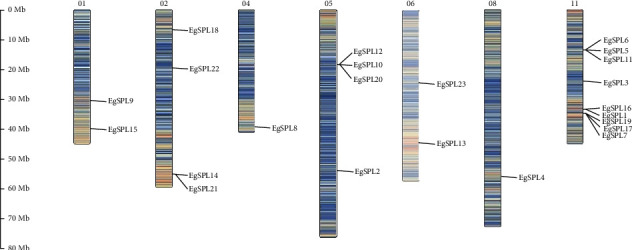
Chromosome location of *EgSPL* genes in *E. grandis* chromosomes. The scale on the left denotes chromosome length in megabases (Mb). Chromosome numbers are indicated by the black numerals at the top of each chromosomal representation. Locations of *EgSPL* genes are highlighted in red text along the chromosomes. The gradient of color from blue to red represents the relative gene density, with warmer colors corresponding to regions of higher *EgSPL* gene density.

Based on the *E. grandis* genome data, chromosome position information was obtained. Twenty-three *EgSPL* genes are unevenly distributed on seven chromosomes (Figure 3). Three sets of genes were found in clusters on Chromosome 11 (*EgSPL5/6/11*, *EgSPL1/16*, and *EgSPL7/17/19*), and a set of genes were found in clusters on Chromosome 05 (*EgSPL10/12/20*) (Figure 3). This suggests that clustering may enhance their functional capabilities.

**Figure 4:**
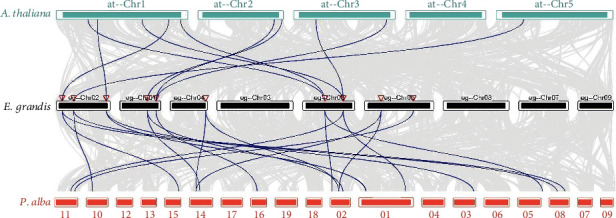
Collinearity analysis between *E. grandis* with *A. thaliana* and *P. alba*. The horizontal bars represent the chromosomes of each species, labeled at the top of *A. thaliana* (Chr1 to Chr5), in the middle for *E. grandis* (Chr1 to Chr11), and at the bottom for *P. alba* (Chr01 to Chr 19). Syntenic relationships are indicated by blue lines connecting the chromosomes, suggesting evolutionary conservation of gene order between the species.

The synteny analysis conducted on *A. thaliana*, *E. grandis*, and *P. alba* revealed that the *EgSPL* genes exhibited a conserved collinear relationship with homolog genes within *A. thaliana* and *P. alba*. Significantly, the assortments of collinear gene pairs vary among *A. thaliana*, *E. grandis*, and *P. alba*, underscoring the intricacy of their phylogenetic interrelations (Figure 4). There are 10 collinear gene pairs identified between *A. thaliana* and *E. grandis* and 18 pairs between *E. grandis* and *P. alba*, speculating that the disparity and presence of gene clusters on the chromosomes probably caused by gene duplication and rearrangement events throughout evolutionary history. Furthermore, the closer genetic affinity between the *SPLs* of *E. grandis* and *P. alba* indicates a higher degree of conservation in woody plants.

### 3.5. Cis-Element Analysis of EgSPLs Promoters

**Figure 5:**
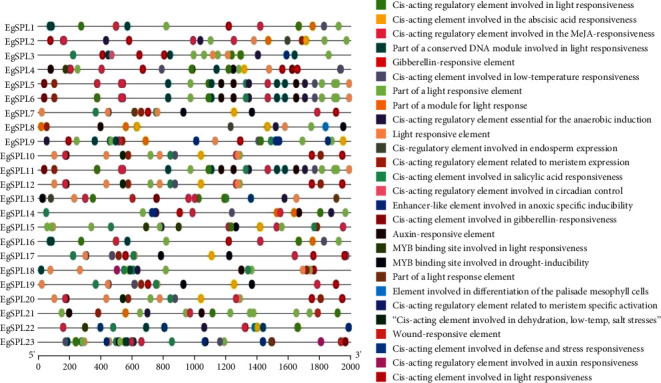
Analysis of homeopathic elements in promoter sequences of EgSPL family 266 genes by PlantCARE. Distribution of homeopathic elements 2000 bp upstream of 267 EgSPL on the left and the type of homeopathic elements on the right.

A total of 27 cis-elements were obtained by PlantCARE from upstream 2 kb sequence of *EgSPLs*, and a large number of cis elements were related to light (9/27); stress (6/27) including low temperature, hypoxia, drought, and defense; growth and development (4/27); and hormone response (7/27) (Figure 5). Hormone-responsive components including ABA, MeJA, auxin, GA, and SA were also identified in promoter of *EgSPL* genes, which was in accordance with important roles in flowering [3], defense response [8], and stress homeostasis of *SPL* genes in previous studies [36]. These cis-elements exist in *EgSPLs*, indicating that the *EgSPL* genes in *E. grandis* may play the similar roles.

### 3.6. Analysis of Tissue Expression Patterns of *SPLs* in *E. grandis* at Different Stages

**Figure 6:**
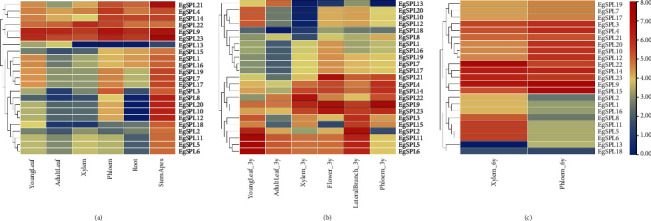
Tissue expression pattern of *EgSPL* family genes. (a) Expression patterns of EgSPL genes in various tissues of semiannual *E. grandis*, including young leaves, adult leaves, xylem, phloem, roots, and stem apex. (b) Expression patterns of *EgSPL* genes in different tissues of 3-year-old *E. grandis*, encompassing young leaves, adult leaves, xylem, flowers, lateral branches, and phloem. (c) Expression patterns of *EgSPL* genes in xylem and phloem of 6-year-old *E. grandis*, highlighting age-related changes.

The gene expression pattern often implied their function. Based on the transcriptome data of *E. grandis*, differential expression patterns of *EgSPLs* were observed across various tissues. Meanwhile, 23 *EgSPL* genes displayed low expression in adult leaves and young roots, while showing high expression in the stem apex and mature xylem and phloem (Figure 6(a)). For instance, *EgSPL5*, *EgSPL6*, and *EgSPL11* showed high expression levels in young leaves, whereas *EgSPL9* and *EgSPL22* were predominantly expressed in xylem. Additionally, *EgSPL23* demonstrated significant expression in both xylem and flowers, alongside *EgSPL9* and *EgSPL21*, suggesting a potential role in the flowering process (Figure 6(b)). In 6-year-old *E. grandis*, *EgSPL9*, *EgSPL22*, and *EgSPL23* were highly expressed in both xylem and phloem. Interestingly, with increasing age, there was an upsurge in the expression of *EgSPL3*, *EgSPL10*, *EgSPL12*, *EgSPL15*, and *EgSPL20* genes in these tissues (Figure 6(c)). This pattern suggests that *EgSPL9*, *EgSPL22*, and *EgSPL23* may play a substantial role in the overall growth and stem development of *E. grandis*.

### 3.7. Analysis of Induced Expression Patterns of *SPLs* in *E. grandis* Under Different Stress

**Figure 7:**
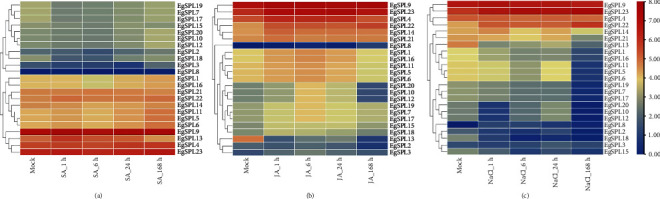
Expression patterns of *EgSPL* genes under (a) MeJA, (b) SA, and (c) NaCl treatment in *E. grandis*.

To further explore the expression patterns of different *EgSPL* family genes under MeJA, SA, and salt stress, transcriptome data were obtained. Under various stress conditions, the *EgSPL* genes exhibit a spectrum of expression profiles, underscoring the multifaceted role in the plant's stress response mechanism. Specifically, under SA treatment, elevated expression levels were recorded for *EgSPL4*, *EgSPL9*, and *EgSPL23*, whereas the rest of *EgSPL*s did not show significant changes. Concurrently, *EgSPL13* showed a short-term increase, whereas *EgSPL7*, *EgSPL17*, *EgSPL18*, and *EgSPL19* demonstrated a short-term decrease (Figure 7(a)). Under MeJA treatment, *EgSPL13* exhibited a decrease expression, contrasting with the general increase observed in other *EgSPL* genes. This differential response suggests intricate regulatory mechanisms in MeJA treatment, potentially reflecting the diverse roles of these genes in MeJA-mediated pathway in *E. grandis* (Figure 7(b)). Under salt stress conditions, a majority of *EgSPL* genes exhibited a decrease expression, either in the short or long term. Conversely, *EgSPL8*, *EgSPL14*, *EgSPL22*, and *EgSPL23* showed a short-term increase expression, which suggests that these genes could play crucial roles in plant adaptation salt stress, highlighting their potential importance in stress response mechanisms (Figure 7(c)). Differently, *EgSPL9* consistently maintained higher expression levels.

### 3.8. Prediction of Protein Structure

**Figure 8:**
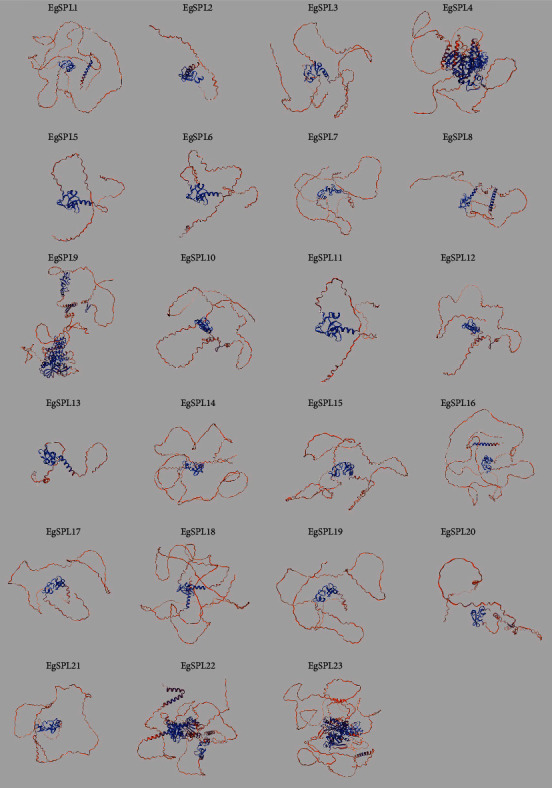
Three-dimensional structure of *EgSPL* predicted by the SWISS model. The *α*-helix and *β*-sheet regions are shown in blue and red, respectively.

In order to study the function of the protein, we predicted the structure of *EgSPLs* from the SWISS-MODEL website. The results showed that these proteins had certain similarities overall, suggesting that they may have similar functions or originate from the same protein family (Figure 8). In addition, the tertiary structures of *EgSPL4*, *EgSPL13*, and *EgSPL23* contain multiple functional domains and show high conservation. In comparison, *EgSPL1-3* and *EgSPL5-12* are mainly random coils, containing a small amount of *α*-helix and *β*-sheet structures, and the overall structure is relatively simple. *EgSPL9* and *EgSPL18* exhibit moderately complex tertiary structures. Overall, EgSPL proteins have high conservation with small structural diversities. These results provide important basis for further functional research and evolutionary analysis.

## 4. Discussion

The completion of genome sequencing of *E. grandis* [37] enabled researchers to study the classification, evolutionary characteristics, and functional prediction of gene families at the genome level. Twenty-three *EgSPL* genes were identified, and then, their gene and protein character, phylogenetic relationships, collinearity, and expression patterns were performed. This provides a basis for in-depth research into the functional roles of *EgSPL* genes in *E. grandis*.

Phylogenetic and evolutionary analysis showed that the *SPLs* of *E. grandis* are closely related to *A. thaliana*, and there are some orthologous genes. Notably, *EgSPL22* and *AtSPL7* are orthologous genes, and *EgSPL22* is highly expressed in xylem and phloem. Previous studies have shown that *AtSPL7* played a major part in copper homeostasis and cadmium stress [25, 26], suggesting that *EgSPL22* may also be involved in these processes and contribute to the development of vascular tissue. Additionally, *EgSPL3* and *EgSPL15* are on the same branch with *AtSPL9* and *AtSPL15*, with high expression in stem apex and lateral branches, which might also mirror their roles in leaf growth regulation and flowering, as well as defense and immunity [14]. Furthermore, *EgSPL14*, positioned alongside *AtSPL10* and *AtSPL11* known for their involvement in nutrient transduction to reproductive growth [11], may serve a comparable function. In *A. thaliana*, *SOC1* regulates *AtSPL3*, *AtSPL4*, and *AtSPL5* by directly binding to the promoter, thereby integrating photoperiodic signals to promote flowering [15]. Interestingly, these genes are all part of the phylogenetic Group IV. Correspondingly, several EgSPL genes in *E. grandis*, particularly *EgSPL7*, *EgSPL17*, *EgSPL19*, *EgSPL21*, *EgSPL1*, *EgSPL16*, and *EgSPL18*, are also classified in this same subgroup and have higher expression in flower and stem apex. Given the close phylogenetic relationship to *AtSPL3/4/5* and the abundance of light-responsive elements in their promoters, these *EgSPL* genes in *E. grandis* are likely to have similar functions in photoperiodic control, potentially influencing flowering and other light-dependent processes.

*MiR156*, which is known for its highly conserved role across the plant kingdom, and its target genes *SPL* are considered to be the main regulators of plant growth and development [38, 39] including mediating crucial pathways related to flowering and aging [29]. This mediation occurs either through the modulation of transcription in downstream target genes or by engaging in diverse biological processes via protein interactions [40–43]. In *A. thaliana*, 11 out of 17 *SPL* genes are regulated by *miR156*. In this study, of the 23 *EgSPL* genes identified in *E. grandis*, 16 *EgSPL* genes were found to contain *miR156* binding sites. Notably, these genes exhibited elevated expression levels in specific tissues such as the stem apex, flower, and xylem, indicating a potential tissue-specific regulatory role. The concurrent presence of *miR156* binding sites in these highly expressed genes suggests a possible intricate interaction with *miR156*, potentially modulating spatial and developmental gene expression within these key tissues. Notably, *EgSPL9* and *EgSPL23* showed high expression levels in various tissues and under hormone-induced conditions and are not regulated by *miR156*. This suggests their significant involvement in distinct developmental processes of *E. grandis*.

The analysis of gene expression patterns facilitates the identification of key *EgSPLs*, which are instrumental in mediating plant responses to both biotic and abiotic stresses. Given the observed upregulation of *EgSPL9*, *EgSPL13*, and *EgSPL23* under SA stress, coupled with the presence of SA-responsive cis-regulatory elements in their promoters, it is plausible to hypothesize that these genes may play a crucial role in the SA-mediated pathways. Similarly, the increased expression of *EgSPL9*, *EgSPL23*, *EgSPL4*, *EgSPL14*, *EgSPL21*, and *EgSPL22* under MeJA stress, in conjunction with the presence of MeJA-responsive cis-regulatory elements in their promoters, suggests a potential involvement in MeJA-mediated pathways. Interestingly, under SA treatment, *EgSPL13* exhibited a transient increase in expression, whereas its expression decreased under MeJA treatment, accentuating its potential role in the SA-mediated pathway. Further experimental validation is necessary to confirm the functional implications of these observations.

## 5. Conclusion

This study identified 23 *SPL* genes of *E. grandis* through bioinformatics analysis and analyzed their physical and chemical properties, evolutionary tree relationships, conserved domains, promoter elements, tissue-specific expression, and hormone stress expression. *EgSPL9*, *EgSPL22*, and *EgSPL23* genes were found to have strong specific expression, and their functions were predicted by a comprehensive phylogenetic tree and promoter functional elements, which are of guiding significance for future generations to study the light response, growth and development, and stress resistance of *E. grandis*.

## Figures and Tables

**Table 1:** Physiochemical properties of the *SPLs* in *E. grandis*.

**Name**	**Sequence ID**	**Length (aa) no. of amino acids**	**MW (kD) molecular mass**	**pI isoelectric points**	**Instability index**	**Grand average of hydropathicity**	**miR156 target site**	**Subcellular localization**
EgSPL1	Eucgr.K02545.2.v2.0	551	59.58332	6.83	50.07	−0.48	+	Cytoplasm nucleus
EgSPL2	Eucgr.E03260.1.v2.0	147	16.97751	6.00	88.37	−1.44	+	Nucleus
EgSPL3	Eucgr.K01828.1.v2.0	376	39.87973	9.12	59.39	−0.75	−	Nucleus
EgSPL4	Eucgr.H04114.2.v2.0	869	97.26905	5.92	50.80	−0.44	+	Cytoplasm nucleus
EgSPL5	Eucgr.K01046.2.v2.0	189	21.07562	9.21	67.96	−1.12	+	Nucleus
EgSPL6	Eucgr.K01046.1.v2.0	236	26.05625	8.66	64.51	−0.78	+	Nucleus
EgSPL7	Eucgr.K02708.2.v2.0	363	39.80499	8.96	55.21	−0.76	+	Nucleus
EgSPL8	Eucgr.D02505.1.v2.0	348	38.35489	8.45	64.98	−0.64	+	Nucleus
EgSPL9	Eucgr.A01019.1.v2.0	983	109.05482	5.79	56.97	−0.41	−	Nucleus
EgSPL10	Eucgr.E01600.1.v2.0	367	38.75971	9.07	72.79	−0.64	−	Nucleus
EgSPL11	Eucgr.K01046.3.v2.0	178	19.94519	9.16	71.57	−1.23	+	Nucleus
EgSPL12	Eucgr.E01600.2.v2.0	321	34.08069	8.96	65.75	−0.62	−	Nucleus
EgSPL13	Eucgr.F03303.1.v2.0	186	21.27769	8.86	69.09	−1.28	+	Nucleus
EgSPL14	Eucgr.B03500.1.v2.0	488	53.53203	8.67	53.61	−0.66	+	Cytoplasm nucleus
EgSPL15	Eucgr.A02441.1.v2.0	390	41.27372	9.12	54.92	−0.61	+	Nucleus
EgSPL16	Eucgr.K02545.1.v2.0	551	59.58332	6.83	50.07	−0.48	+	Cytoplasm nucleus
EgSPL17	Eucgr.K02708.3.v2.0	315	34.66033	9.41	62.03	−0.78	+	Nucleus
EgSPL18	Eucgr.B00631.1.v2.0	524	57.17946	8.74	55.34	−0.64	+	Nucleus
EgSPL19	Eucgr.K02708.1.v2.0	363	39.80499	8.96	55.21	−0.76	+	Nucleus
EgSPL20	Eucgr.E01600.3.v2.0	257	26.71659	7.37	59.71	−0.43	+	Nucleus
EgSPL21	Eucgr.B03518.1.v2.0	318	34.53342	9.50	53.22	−0.68	+	Nucleus
EgSPL22	Eucgr.B01228.1.v2.0	821	91.22122	5.77	55.35	−0.42	−	Cytoplasm nucleus
EgSPL23	Eucgr.F01828.1.v2.0	1078	119.28528	7.11	58.38	−0.53	−	Nucleus

*Note:* In the penultimate titled “*miR156* Target Site,” the presence of a *miR156* target site within the SPL protein sequence is indicated by a plus sign (+), while its absence is denoted by a minus sign (−).

## Data Availability

All data generated or analyzed during this study are included in this article.
